# Internet Public Opinion Evolution in the COVID-19 Event and Coping Strategies

**DOI:** 10.1017/dmp.2020.299

**Published:** 2020-08-12

**Authors:** Zufeng Zhong

**Affiliations:** Business School, Lingnan Normal University, Zhanjiang, China and Guangdong Coastal Economic Belt Development Research Center, Zhanjiang, China

**Keywords:** COVID-19, Internet public opinion, latent Dirichlet allocation (LDA), public health emergency, sentiment analysis

## Abstract

**Objectives:**

In this study, we carried out a text analysis on the information disseminated and discussed among netizens on the Baidu Post Bar (the world’s largest Chinese forum) during the coronavirus disease 2019 (COVID-19) epidemic, to create a policy basis for health administrative departments.

**Methods:**

We used Python tools to search for the relevant data on the Baidu Post Bar. Next, a text analysis was performed on the posts’ contents using a combination of latent Dirichlet allocation (LDA), sentiment analysis, and correlation analysis.

**Results:**

According to the LDA analysis, the public was highly interested in topics such as COVID-19 prevention, infection symptoms, infection and coping measures, sources of transmission and treatments, community management, and work resumption. The majority of the public had negative emotional values, yet a portion of the public held positive emotional values. We also performed a correlation analysis of the influencing factors was established.

**Conclusions:**

Netizens’ degree of concern shown in their posts was greatly associated with the spread of COVID-19. With the rise, diffusion, outbreak, and mitigation of COVID-19 in China, netizens have successively created a large number of posts, and the topics of discussion varied over time. Therefore, the media and the government have the responsibility to distribute positive information, to correctly guide the public’s emotions to bring some sort of reassurance to the public.

Notable achievements have been made in global infectious disease prevention and control. However, infectious diseases remain the major diseases harming mankind. Specifically, the emergence and spread of dozens of new infectious diseases worldwide, including the acquired immunodeficiency syndrome (AIDS), H7N9 avian influenza, Ebola virus, and Middle East respiratory syndrome (MERS), have become major global public health emergencies. In December 2019, the coronavirus disease 2019 (COVID-19) broke out. On February 28, 2020, the World Health Organization (WHO) began to give daily updates of the COVID-19 situation, and regional and global risk levels were raised to the highest level (“extremely high”). On March 11, the WHO evaluated that the current COVID-19 epidemic and referred to it as a global pandemic. As reported by the WHO as of April 10, 2020, there were altogether 1,610,909 people infected with the COVID-19, with a death toll of 99,690 cases.

Along with the rise of the Internet, China has seen the emergence of a new population actively engaged in using this technology—citizens popularly and officially referred to as netizens.^1^ Major public health emergencies tend to arouse heated discussions among netizens. Such critical posts reflect the changes of the netizens’ emotions (either positive or negative), and some negative emotions even induce group panic and propagate rumors. Consequently, emotions play a vital role in the information diffusion process during major public health emergencies. Existing studies have used Web texts to predict^2-4^ and monitor^5-8^ public health emergencies. Typically, artificial intelligence (AI) is mainly used to monitor online information propagation rules,^9-11^ and analyze the influencing factors of online Web texts and infectious disease transmission.^12-14^ The occurrence of a major public health emergency breaks the public’s original psychological balance, which results in a psychological abnormality. As a result, identifying the emotions^15^ of netizens can be of tremendous help in promoting public health prevention^16^ and health education.^17,18^

Finding ways to guarantee the safety of the public and limit risks of transmission among the public during public health emergencies is an important consideration for organizers and managers. China has taken measures to control the COVID-19 outbreak at an early stage and has managed to mitigate its effects. We analyzed the data published by means of the Baidu’s Post Bar, with a focus on the sentiment analysis of the emotional tendency in the text contents of the Post Bar, to enhance the automatic analysis capacity for Post Bar texts and reduce the difficulty in public opinion monitoring. An understanding of the public’s opinion not only helps facilitate the prevention and control of COVID-19 but also contributes to social stability and harmony.

## METHODS

### Data Source

The data source of this study is Baidu Post Bar (text language is Chinese form). As of its release on the market in 2013, the Baidu Post Bar, an independent brand developed by Baidu, has become the world’s largest Chinese online community. The Baidu Post Bar is in reality a keyword-based subject communication community. Closely related to the search engine, the Baidu Post Bar creates a free Internet space where Chinese netizens can freely share their views and opinions with like-minded peers. Currently, the Baidu Post Bar has a dense population of Chinese netizens, with a total registered user group of 1500 million, over 22 million posts, 3.5 billion subjects, and 64.6 billion messages.

### Data Collection

We designed Web crawlers based on the re module of Python, conducted the approximate string matching of the text strings, and extracted the appropriate strings from Web pages. Thereafter, we crawled all post data in the “coronavirus bar” and “COVID-19 bar” forums. The major crawled fields included the user name, user nickname, main post name, post name, post time, and post content. Within the collected data, a total of 8846 users participated in the discussion, resulting in 3042 topics and 31,587 posts. The data were cleaned, and this process included the elimination of irrelevant advertising posts and meaningless posts. Then, the posts between January 1, 2020, and April 10, 2020 were selected. Finally, 15,800 posts were retained as the data source for this work.

### Data Cleaning

Subsequently, the post contents irrelevant to this study were eliminated, such as advertising messages, abnormal information, pictures, and videos. The basic information in the post-bar, such as the user name and post time, were recorded, and then the posts’ contents were classified and summarized in chronological order.

### Segmentation

The sorted texts were subject to wrongly written character correction, emoticon elimination, and the removal of terms with no specific meaning. The contents were segmented using the cut function in the jieba library of Python, to provide the basis for subsequent research on topic modeling and sentiment analysis. The Python jieba segmentation kit is widely recognized as a useful word segmentation tool in Chinese text preprocessing (https://pypi.org/project/jieba/). Based on the highly efficient word-graph screening function in the Trie structure, jieba word segmentation is capable of generating sentences where all the Chinese characters are involved in a directed acyclic graph. It also checks the maximum-probability path and word frequency-based maximum segmentation combination through dynamic planning.

### Word Frequency Analysis

Word frequency analysis is a representative text content analysis method that determines the hotspot and variation trend based on changes in the word occurrence frequency. The counter function in Python was implemented to calculate the occurrence frequency of each word after segmentation. The words were sorted from the highest frequency to the lowest one.

### Latent Dirichlet Allocation

Latent Dirichlet Allocation (LDA) was proposed by Blei et al. in 2003 to identify the topic distribution of a document.^19^ It can present the topics in each document of a folder similarly to the manner it is displayed in a probability distribution. As a result, it allows for topic clustering or text classification based on the topic distribution, after the topic distribution is extracted from documents.^20,21^ In this study, we used the LDA library of the Python tool to carry out the LDA on the processed Chinese texts (the results of this LDA process are translated into English), with the following parameters set: alpha = 1.25, beta = 0.1, and K = 8.

### Sentiment Analysis

Text sentiment analysis is also called opinion mining and opinion analysis. It refers to the process of analyzing, processing, concluding, and reasoning the subjective texts with emotional colors.^22-25^ We integrated the SnowNLP in the Python tool for the sentiment analysis of the text in each post (github.com/isnowfy/snownlp). SnowNLP is a Python library that can transact Chinese texts and perform sentiment analysis on Chinese sentences. The value of the sentiment analysis indicates the probability of the sentence representing a positive emotion, with a range of (0,1). Typically, a value closer to 1 suggests that the expressed emotion is more positive, while a value closer to 0 suggests that the expressed emotion is more negative.

### Correlation Analysis

Correlation analysis is a statistical analytic method used to investigate the correlation between 2 or more random variables with equal status, where the discussed variables share an identical status, and the analysis focuses on the correlation features between the random variables.^26,27^ We used the corr() function in Python to establish a correlation analysis on the related influencing factors for the posts on the Baidu Post Bar during the COVID-19 transmission process.

## RESULTS

### Data Analysis

**FIGURE 1:**
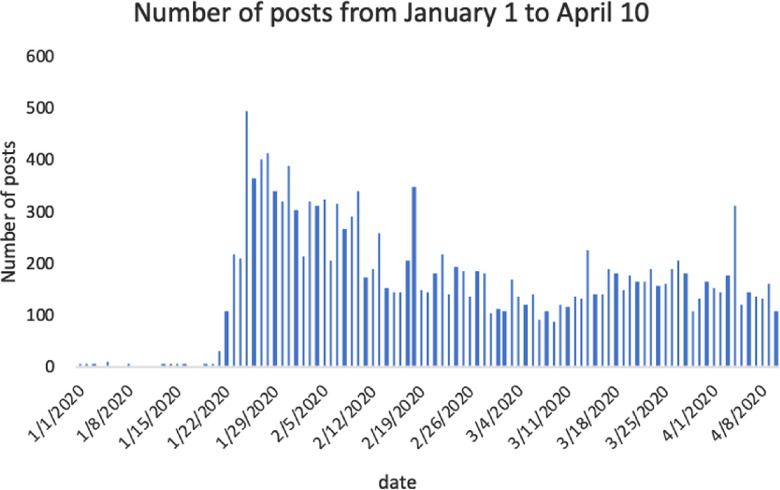
Number of Posts Published on a Daily Basis.

The posts published between January 1, 2020, and April 10, 2020, were organized based on the date. As illustrated in Figure 1, before February 22, 2020, netizens paid little attention to COVID-19. This can be explained by the few number of confirmed cases initially reported when the COVID-19 broke out in Wuhan (Hubei province, China), which did not grab the public’s attention, thus justifying the low number of related discussions. Later, with the exponential increase in the number of confirmed cases, COVID-19 gradually captivated the public’s attention and discussions.

### Analysis of the “Hottest” Posts

The top 5 posts with the highest number of replies are shown in Table 1. Table 1 shows the posts with the highest attention were concentrated at the end of January. At this time, COVID-19 was still in the outbreak period, and the public was in a period of panic. Hence, the topics in those posts essentially consisted of discussions on surgical mask purchasing (January 23), attention on vaccine development (January 25), and posts for encouragement (January 28). Additionally, there was another important date in March (March 3), which was generated by the release of the real-time records of a patient infected with COVID-19, which also attracted wide attention from the netizens.

**TABLE 1:** Top 5 Posts

Topic	Number of Replies	Post Time
Excuse me, can COVID-19 be prevented by a facemask respirator?	159	2020/1/23
I’ve always wanted to write a post to record the course of my illness	102	2020/3/3
Fight the coronavirus together	86	2020/1/28
How long do you think it will be before the vaccine is developed?	84	2020/1/25
Update on the latest news	83	2020/1/25

### User Statistics

**FIGURE 2:**
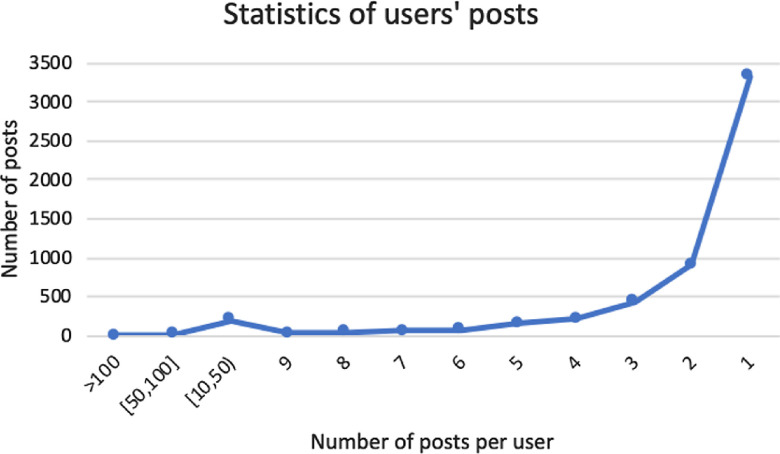
Number of Posts From Each User.

The users’ posts are statistically organized in Figure 2. The number of posts from each user ranged from 0 to10, indicating that most users predominantly read and acquired information, while few users actively shared information. The user with the highest number of posts had created a total of 421 posts, while the topics and contents of these public posts mainly involved preventive measures against the COVID-19 and ways to enhance the body’s immune system.

### LDA

**FIGURE 3:**
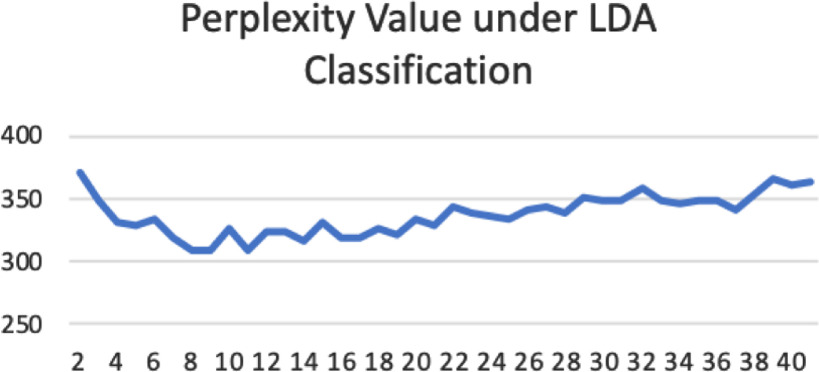
Perplexity Value Under the LDA classification.

The first step was to determine the number of categories to be divided by the LDA. The perplexity attribute in Python sklearn.decomposition (https://scikit-learn.org/dev/index.html) was used to identify the number of LDA classifications (minimum perplexity corresponds to a superior number of classifications). The value of the perplexity was counted by setting the classification as 2-40. As illustrated in Figure 3, the perplexity reaches its minimum with optimal performance when the classification is equal to 8. Thus, the research was performed with an LDA classification in the condition of K = 8 (where K denoted the number of categories). After we set the parameter, a text analysis was conducted on the posts’ contents, which revealed that the posts’ contents could be classified as the 8 topics presented in Table 2.

**TABLE 2:** Eight Topics According to the Latent Dirichlet Allocation (English Version)

	Topic	High Frequency Words
Topic 1	Prevention of COVID-19	Virus, mask, reply, epidemic situation, coronavirus, Wuhan, pneumonia, Infected, hospital, U.S.A., novel coronavirus, China, country, symptom, quarantine, case, feel, testing, Chinese medicine
Topic 2	Effect of TCM on COVID-19	Virus, reply, mask, epidemic situation, pneumonia, China, novel coronavirus, infected, chinese medicine, patient, human body, hospital, Wuhan, U.S.A., coronavirus, quarantine, case
Topic 3	Infection of COVID-19	Virus, epidemic situation, mask, hospital, pneumonia, patient, China, Wuhan, reply, coronavirus, diagnosis, symptom, new type, infected, spread, novel coronavirus, case
Topic 4	Death of patients diagnosed with COVID-19	Epidemic situation, virus, mask, reply, novel coronavirus, pneumonia, country, hospital, Wuhan, China, coronavirus, U.S.A., infected, patient, situation, diagnosis, death
Topic 5	Comparison of the epidemic situation between China and the U.S.A.	Epidemic situation, novel coronavirus, China, reply, mask, pneumonia, infected, Wuhan, quarantine, U.S.A., symptom, hospital, coronavirus, human beings, diagnosis, healthy
Topic 6	Isolation policy, infection and death toll in China and the U.S.A	Virus, reply, mask, novel coronavirus, epidemic situation, pneumonia, diagnosis, China, Infected, coronavirus, treatment, U.S.A, country, really, quarantine, doctor, death
Topic 7	Symptoms and origin of COVID-19	China, epidemic situation, infected, novel coronavirus, hospital, Wuhan, symptom, mask, coronavirus, pneumonia, quarantine, U.S.A, treatment, 1 kind, country, find, feel, human beings
Topic 8	China’s help to other countries	Virus, mask, reply, epidemic situation, infected, China, patient, pneumonia, novel coronavirus, Wuhan, U.S.A., quarantine, coronavirus, hospital, expert, Italy, protect

As delineated in Table 2, the 8 main topics were centralized on ways to properly use a mask and other instruments to curb the spread of the COVID-19 pandemic; some netizens were interested in the epidemic situation in Wuhan (Hubei Province, China) and the therapeutic effects of traditional Chinese medicine therapy. Some gave information on the COVID-19 infection and its associated death toll. In the later stages of the epidemic in China, some compared the circumstances of those in Wuhan with those in America, including the quarantine measures put in place by the Chinese and American governments, as well as the number of suspected cases and mortalities. Furthermore, they also concentrated on how Chinese experts aided foreign countries, including America and Italy, in the fight against the pandemic. Some dug into the origin of the epidemic based on the symptoms of the virus. All of these subjects are related to every aspect of the COVID-19 infection process, which revealed the public’s concern about the spread of the COVID-19 virus, and their strong interest in the prevention, treatment, and assistance to other countries in the battle against the disease.

### Sentiment Analysis

**FIGURE 4:**
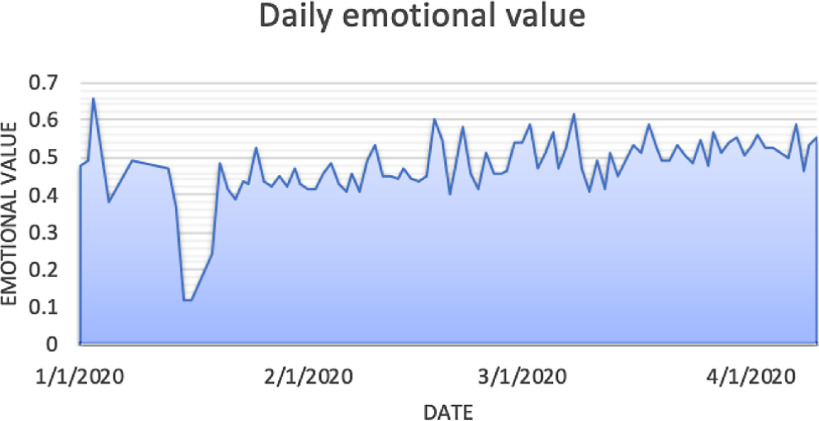
Emotional Values According to Different Dates.

A positive and negative emotion analysis was performed on the post texts in chronological order. In this study, the mean() function was used to process the sentiment analysis results, as depicted in Figure 4. Most values were between [0.4,0.5], suggesting that the post-bar users mostly held negative emotions during the COVID-19 epidemic period. The emotion value was the lowest on January 15, which was due to the fact that only a few posts were released on that day, and the posts’ contents included the introduction of the effects of pharmacologic prevention on the virus, leading to the low emotional value.

The emotion value was the highest on January 3, which was due to the fact that the number of netizens posted on this date is less, and they are blindly optimistic. The content of the posts is that the COVID-19 is not a serious illness, will soon pass. High emotional values were obtained on February 20, February 26, March 5, March 11, and March 20. February 20 and February 26 were the dates of the announcement of the Wuhan lockdown and nationwide social distancing, which induced a heated debate among the netizens, who had confidence in overcoming the virus. On March 5 and March 11, the netizens principally posted to express their appreciation and gratitude toward all medical staff from all over the country who rushed to rescue the citizens of Wuhan, and so the posts’ contents were full of positive information. By March 20, the COVID-19 epidemic in Wuhan was basically under control, the medical staff had begun to return to their positions, various labor sectors resumed their operations, and the public expressed their joyous emotions over the success in the virus’s prevention and control.

### Correlation Analysis

From January 11, 2020, to April 10, 2020, the daily total number of infected people in China, the daily number of newly infected people, the daily total number of deaths, and the daily number of new deaths (Table 3) were selected, with which a correlation analysis of the daily emotional value (the missing values were filled with the averages) and the number of posts (the missing value was filled with the value on the previous day) was performed.

**TABLE 3:** Correlation Analysis

	Daily number of newly infected people	Daily total number of infected people	Daily number of new deaths	Daily total number of deaths	Daily emotional value	Daily post number
Daily number of newly infected people	1	−0.08023		−0.22624	−0.11847	0.390235
Daily total number of infected people		1	0.173644		0.448322	0.149528
Daily number of new deaths			1	−0.0425	−0.03227	0.385696
Daily total number of deaths				1	0.465471	0.072502
the daily emotion value					1	0.111679
Daily post number						1

As demonstrated in Table 3, the daily total number of infected people index was the value most significantly correlated with the daily total number of deaths index; it was followed by the correlation between the daily number of newly infected people index and the daily number of new deaths index, with a correlation coefficient as high as 0.74. Such results confirmed that the number of infected people was positively correlated with the number of deaths due to COVID-19. Also, the daily total number of infected people index (0.48) and the daily total number of deaths index (0.46) were highly correlated with the daily emotional value, indicating that the public’s emotions fluctuated with the outbreak and evolution of the COVID-19 epidemic. The daily number of newly infected people index (0.39) and the daily number of new deaths index (0.38) were remarkably correlated with the daily number of posts, revealing that the number of daily newly infected people and the number of deaths instigated hot debates among the netizens. Netizens tended to give positive comments when the number of confirmed cases and deaths increased, which is due to the fact that more netizens were focused on the matter when more people become infected, encouraging one another reciprocally.

## DISCUSSION

We used the phrase “COVID-19 outbreak” as the object of study, performed a statistical analysis of the comments in the Baidu Post Bar, and analyzed the time sequences. Additionally, we also established the influencing factors for the online propagation of information on the COVID-19 epidemic in terms of the post popularity, user analysis, LDA, and sentiment analysis. The research results revealed the following. (1) The diversity, simplicity, and rapidity of online propagation modes encourage the public to acquire and share information online and express their opinions and emotions toward an event. (2) The information released online is highly correlated with the evolution of the event in question. For instance, the focus of the public discussions changed with the genesis, development, outbreak, and control of the COVID-19 epidemic. (3) The discussions among the public on COVID-19 involved multiple aspects, including the source, prevention, and treatment of the COVID-19, as well as the attention and encouragement provided to the medical staff and patients. (4) During the COVID-19 outbreak, most comments published by netizens in the Baidu Post Bar contained negative emotions, yet a small number of comments were positive. Such findings indicate that the public held positive emotions to cope with the disaster, even though they abhorred this disastrous event.

According to the research results, we propose the following insights or suggestions for the government’s public administrative department to monitor and regulate the online public emotional transmission in coping with public health emergencies. (1) First and foremost, network information transmission is an important component of public opinion transmission in public health emergencies, which reflects the real opinions and attitudes of all levels of society. It is the “barometer” of social public opinions, which should trigger significant attention from the corresponding media and government authorities. (2) Based on the transmitted contents, information on the COVID-19 should be vigorously promoted, so that the public understands the facts concerning the genesis, development, and evolution of the COVID-19 epidemic. (3) Starting from the subject of communication, more attention should be paid to guiding the attitudes and emotions of important users with opinion leader influence, thus enhancing the overall grasp of public opinions. (4) The government should take into consideration the emotions of the public to avoid the propagation of negative social influences, resulting in a massive panic. Appropriate negative and positive emotions both play a beneficial role in the prevention and control of the epidemic because negative emotions remind netizens to be alert for the disease while positive emotions pacify the mood of netizens. Extreme responses, including extreme negative emotions (facilitating group panic) and extreme positive emotions (leading to overconfidence), generate detrimental impacts on the prevention and control of the epidemic. Hence, the media and the government should preserve the netizens’ right to know and guarantee the transparency of information on the effective treatment modalities so as to eradicate rumors. The shared information should be encouraging and optimistic, to soothe emotions, and inspire the public. Negative emotions should be addressed in a timely manner to effectively ensure the benign development of public opinion.

## CONCLUSIONS

We analyzed the data published by means of the Baidu Post Bar, with a focus on the sentiment analysis with respect to the emotional tendencies in the text contents of the Post Bar, aiming to enhance the automatic analytic capacity for Post Bar texts and reduce the difficulty in public opinion monitoring. This work not only facilitates the prevention and control of the COVID-19 epidemic, but also contributes to social stability and harmony.
